# Multiple donor–acceptor design for highly luminescent and stable thermally activated delayed fluorescence emitters

**DOI:** 10.1038/s41598-023-34623-9

**Published:** 2023-05-11

**Authors:** Bhagya Madushani, Masashi Mamada, Kenichi Goushi, Thanh Ba Nguyen, Hajime Nakanotani, Hironori Kaji, Chihaya Adachi

**Affiliations:** 1 Center for Organic Photonics and Electronics Research (OPERA), Kyushu University, Motooka, Nishi, Fukuoka, 819-0395 Japan; 2 Institute for Chemical Research, Kyoto University, Uji, Kyoto 611-0011 Japan; 3 International Institute for Carbon Neutral Energy Research (I2CNER), Kyushu University, 744 Motooka, Nishi, Fukuoka 819-0395 Japan; 4 Present Address: Department of Chemistry, Graduate School of Science, Kyoto University, Sakyo-ku, Kyoto, 606-8502 Japan

**Keywords:** Materials science, Optics and photonics

## Abstract

A considerable variety of donor–acceptor (D–A) combinations offers the potential for realizing highly efficient thermally activated delayed fluorescence (TADF) materials. Multiple D–A type compounds are one of the promising families of TADF materials in terms of stability as well as efficiencies. However, those emitters are always composed of carbazole-based donors despite a wide choice of moieties used in linearly linked single D–A molecules. Herein, we developed a multiple D–A type TADF compound with two distinct donor units of 9,10-dihydro-9,9-dimethylacridine (DMAC) and carbazole as the hetero-donor design. The new emitter exhibits high photoluminescence quantum yield (PLQY) in various conditions including polar media blend and high concentrations. Organic light-emitting diodes (OLEDs) showed a reasonably high external quantum efficiency (EQE). In addition, we revealed that the multiple-D–A type molecules showed better photostability than the single D–A type molecules, while the operational stability in OLEDs involves dominant other factors.

## Introduction

Thermally activated delayed fluorescence (TADF) has emerged as the third-generation emitter in organic light-emitting diodes (OLEDs), which facilitates harvesting both singlet and triplet excitons through reverse intersystem crossing (RISC), resulting in 100% internal quantum efficiency (IQE)^1^. This phenomenon occurs at room temperature because of a small single-triplet energy gap (Δ*E*_ST_) between the lowest excited singlet and triplet states (S_1_ and T_1_), which can be realized by spatially separating the highest occupied molecular orbital (HOMO) and the lowest unoccupied molecular orbital (LUMO). Thus, the unique molecular design of TADF emitters is based on various types of donor (D) and acceptor (A) units^2–5^. Recent interest in the research area of TADF is related to the control of rate constants, for example, maximizing the RISC rate (*k*_RISC_)^6–14^. The fast *k*_RISC_ can reduce long-lived triplet excitons, which would contribute to decreasing exciton losses and efficiency rolloff^15,16^. Since fluorescent emitters generally exhibit better stability than phosphorescence and TADF emitters with high energy triplet excitons, another expected benefit of the fast *k*_RISC_ is the improvement of the device durability, which has been a long-standing and serious problem in the applications of TADF^17–20^. However, the relationships among device durability, *k*_RISC,_ and molecular structures have not been understood comprehensively. For example, a series of compounds with carbazole donors and benzonitrile acceptors represented by 4CzIPN achieved satisfactory high stabilities despite moderately fast *k*_RISC_ (~ 10^6^ s^−1^)^1,21^. From the viewpoint of the chemical structure, the multiple donor units allow not only variation of D–A interactions and charge transfer (CT) strength but also the intramolecular π–π interactions and delocalization effect, which may be associated with the high stability^22^. In addition, the hetero-donor strategy introducing second donor units might improve stabilities further in addition to photophysical properties^23,24^. However, the multiple and hetero-donor designs are always based on carbazole-based donors such as parent carbazole and 3,6-disubstituted-carbazole^25–29^. Therefore, it is desired to verify multiple and hetero-donor strategies by incorporating a different type of donor and compared to linearly linked single D–A molecules.

**Figure 1:**
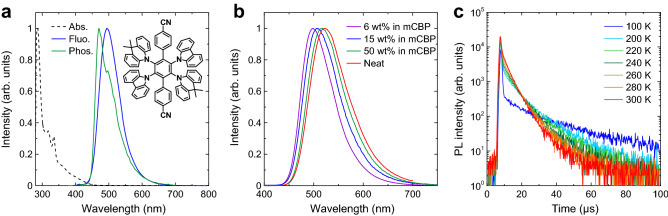
(**a**) Absorption and fluorescence spectra at room temperature, and phosphorescence spectrum at 77 K for 2Cz2DMAC2BN in toluene. Inset: Chemical structures of 2Cz2DMAC2BN. (**b**) PL spectra for 2Cz2DMAC2BN doped films of mCBP and neat 2Cz2DMAC2BN film. (**c**) Temperature dependence of transient PL decay for 6-wt%-2Cz2DMAC2BN doped film of mCBP.

In this paper, we developed a new multiple and hetero-donor type molecule, named 2Cz2DMAC2BN (Fig. 1, inset), consisting of two carbazole (Cz), two 9,10-dihydro-9,9-dimethylacridine (DMAC), and two benzonitrile (BN) units connected to the center phenyl ring. The DMAC-based emitters form strong CT because of the stronger donor nature of DMAC compared to Cz and the more twisted structure. By combining with the weak acceptor moiety, the emission colors of 2Cz2DMAC2BN appear from sky blue to green, making varieties of choice of reference emitters possible. In addition, high photoluminescent quantum yield (PLQY) and small Δ*E*_ST_ prove efficient TADF properties for 2Cz2DMAC2BN. Thus, the stabilities of some different types of excellent TADF materials with similar photophysical properties were accordingly compared. The analysis of the photophysical parameters and stabilities of these compounds would provide a better understanding of the structure–property relationship in multiple and hetero-donor designs.

## Results and discussion

### Synthesis

The compound was easily synthesized from commercially available 1,4-dibromo-2,3,5,6-tetrafluorobenzene in three steps with the Suzuki coupling followed by S_N_Ar reaction (Supplementary Methods S1)^29^. The deprotonated DMAC in the presence of sodium hydride attacked the 2,5-position of tetrafluoro derivatives. The relatively bulky DMAC moieties prevent additional substitutions even in the excess amounts of nucleophiles. Thus, the following S_N_Ar using carbazole *N*-anion forms the target compound in good yields. The product was carefully purified by column chromatography, recrystallization and vacuum sublimation. The molecular structure was confirmed by X-ray single crystal analysis (Supplementary Fig. S1). The crystal contains two independent molecules with an inversion center, where the dihedral angles between DMAC and center phenyl (Ph) rings are varied from 77° to 90°. Since the calculated angle is 79° (Supplementary Fig. S2), the packing effect might cause the highly twisted structures. This result suggests some degree of freedom for the rotation of substituted rings. Since the DMAC-Ph bond is considered to be highly twisted close to 90° in the excited states because of the CT^30^, the molecule should easily form stable geometries in the excited states.

### Photophysical properties

The photophysical characteristics of 2Cz2DMAC2BN were fully characterized in solution and solid states (Fig. 1). A high PLQY of 91% was obtained in toluene, where the small Δ*E*_ST_ of 0.01 eV and fast delayed lifetime (τ_d_) of 3.8 µs were also achieved (Table 1), indicating efficient TADF properties. These properties are comparable to those of 4CzIPN which is one of the best green TADF molecules^1^. The phosphorescence spectrum in toluene at 77 K showed shoulder peaks, indicating that the T_1_ state has a locally excited state (LE) character. The triplet LE (^3^LE) of Cz and DMAC are relatively high at > 3.0 eV^31^. Although the ^3^LE of BN-Ph-BN was reported to be 2.9 eV^29^, the highly twisted DMAC might offer the space to planarize BN-Ph-BN rings, resulting in the decrease of ^3^LE. Interestingly, 2Cz2DMAC2BN showed constantly high PLQY even in polar solvents such as acetone, which is a clear difference from 4CzIPN (Supplementary Fig. S3 and Table S1). This feature suggests a great potential to maintain high PLQY in high doping concentrations including neat conditions, where the TADF molecules having D–A structures may reduce PLQY as the polar media. In addition, the centrosymmetric structure of 2Cz2DMAC2BN leads to low polarity.

**Table 1:** Photophysical characteristics of 2Cz2DMAC2BN in toluene, blend films with mCBP, and neat film.

Condition	λ_PL_ (nm)	S_1_ (eV)	T_1_ (eV)^a^	Δ*E*_ST_ (eV)	Φ_PL_ (–)^b^	Φ_p_/Φ_d_ (–)^b^	τ_p_ (ns)^b^	τ_d_ (µs)^b^	*k*_r_ (10^6^ s^−1^)	*k*_ISC_ (10^7^ s^−1^)	*k*_RISC_ (10^5^ s^−1^)
In toluene	496	2.79	2.78	0.01	0.91	0.20/0.71	46	3.8	4.3	1.7	11.7
In 6 wt% film^c^	498	2.76	2.71	0.05	0.95	0.28/0.67	34	3.6	8.2	2.1	9.2
In 15 wt% film^c^	507	2.75	2.72	0.03	1.0	0.35/0.65	47	3.8	7.5	1.4	7.5
In 50 wt% film^c^	517	2.70	2.64	0.06	1.0	0.42/0.58	55	3.5	7.6	1.1	6.8
In Neat film	522	2.67	2.60	0.07	0.76	0.38/0.38	62	2.0	6.1	1.0	8.1

^a^Measured at 77 K; ^b^Measured under N_2_ in solution or Ar in films; ^c^Doped in mCBP.

In fact, the blend films of 2Cz2DMAC2BN and mCBP (3,3′-di(9*H*-carbazol-9-yl)-1,1′-biphenyl) kept near-unity PLQY in 6–50 wt% doping concentrations (Fig. 1b and Table 1). The emission wavelengths also showed small redshifts. These are advantageous to optimize the OLED because the doping concentrations in the emissive layer (EML) significantly affect charge transport ability. The Δ*E*_ST_ and τ_d_ are comparably good with those in the solution. The temperature dependence of the transient PL decay clearly showed the typical behavior of TADF with the increase of delayed fluorescence (Fig. 1c). The optical properties in other hosts such as CCP (9-phenyl-9*H*-3,9′-bicarbazole) and in PPT (2,8-bis(diphenyl-phosphoryl)-dibenzo[*b*,*d*]thiophene) are also excellent although the polarity of PPT is relatively higher than mCBP (Supplementary Fig. S4 and Table S2)^32^. The calculated *k*_RISC_ is close to 10^6^ s^−1^, which is similar to that of 4CzIPN. The neat film also exhibited good emission properties with a PLQY of 76%, indicating the suppression of the concentration quenching. The emission maximum in the neat film is only 24 nm redshifted from that in a 6 wt%-doped film of mCBP (Fig. 1b). These results adequately demonstrate the efficient emission properties of 2Cz2DAMC2BN.

### OLED device performances

To demonstrate the efficient TADF properties of 2Cz2DMAC2BN, the OLEDs were fabricated according to the report for 4CzIPN^21^. The device structures are ITO (100 nm)/HAT-CN (10 nm)/Tris-PCz (30 nm)/electron blocking layer (EBL) (5 nm)/x-wt%-2Cz2DMAC2BN:host (30 nm)/hole blocking layer (HBL) (10 nm)/BPy-TP2 (40 nm)/Liq (2 nm)/Al (100 nm), where HAT-CN is dipyrazino[2,3-*f*:2′,3′-*h*]quinoxaline-2,3,6,7,10,11-hexacarbonitrile, Tris-PCz is 9,9′,9″-triphenyl-9*H*,9′*H*,9″*H*-3,3′:6′,3″-tercarbazole, BPy-TP2 is 2,7-bis(2,2′-bipyridine-5-yl)triphenylene, and Liq is 8-hydroxyquinolinolato-lithium (Supplementary Fig. S5). The devices with mCBP and CCP hosts have the EBL of the neat layer of each host material and the HBL of T2T (2,4,6-tris(biphenyl-3-yl)-1,3,5-triazine). The devices with a PPT host have the EBL of mCBP and the HBL of PPT. The HOMO–LUMO energy levels of 2Cz2DMAC2BN were estimated to be −5.4 and −2.5 eV from the electrochemical measurements, where the redox processes were found to be stable (Supplementary Fig. S6). The HOMO–LUMO energies in the neat film measured by the photoelectron yield spectroscopy and optical energy gap were −5.75 and −3.15 eV. The host materials of mCBP and CCP consisting of Ph and Cz rings are p-type while the PPT host with phosphine oxide is n-type. However, the charge carrier transport properties in the EML would be changed depending on the doping concentrations of 6, 15, and 50 wt% of bipolar 2Cz2DMAC2BN.

**Figure 2:**
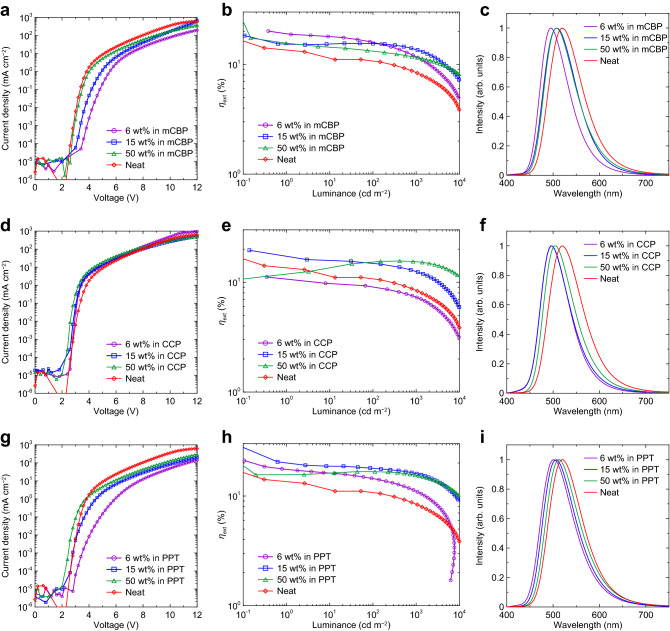
OLED performances for 2Cz2DMAC2BN-based devices. (**a**–**c**) The devices with mCBP host. (**d**–**f**) The devices with CCP host. (**g**–**i**) The devices with PPT host. (**a**,**d**,**g**) Current density–voltage characteristics. (**b**,**e**,**h**) EQE-luminance characteristics. (**c**,**f**,**i**) EL spectra in the OLEDs at 6 V.

The 6 wt%-2Cz2DMAC2BN doped mCBP device showed a reasonably high EQE of 19% although the efficiency rolloff is slightly large (Fig. 2 and Table 2). The theoretical EQE is calculated to be 20% from the estimated 100% carrier balance and exciton utilization efficiency, experimental PLQY of 95%, and simulated outcoupling efficiency of 21% (Supplementary Fig. S7), which is consistent with the experimental result. The current density (*J*)-voltage (*V*) and rolloff characteristics were improved with the increase of the doping concentrations since the recombination zone shifted to the center by enhancing the electron transport in the EML as observed in the devices of 4CzIPN^21^. Although the PLQY values were nearly the same for 6–50 wt% doped mCBP films, the maximum EQE values were slightly decreased because of some quenching processes. The *J*–*V* characteristics of the devices using CCP hosts are independent of the doping concentrations (Fig. 2), indicating that the recombination always happened at the EML/HBL interface. This is due to the markedly higher hole mobility of CCP-host film as confirmed by hole-only and electron-only devices (HOD and EOD) (Supplementary Fig. S8). However, the EQE characteristics for the CCP devices are strongly dependent on the doping concentrations. The 50 wt%-doped CCP device also suggested the existence of the exciton quenching in the high doping concentration, probably because of the increase of the radical anion state of 2Cz2DMAC2BN caused the annihilation. On the other hand, the electron transport in the guest 2Cz2DMAC2BN might be small in the devices using an n-type PPT host. Although the low doping concentration of 6 wt% showed relatively large rolloff because of poor hole mobility, the 15 wt%-doped PPT device showed better rolloff characteristics and overall EQE than the p-type devices. Interestingly, the device operational lifetime of PPT device is better than those of mCBP and CCP devices (Supplementary Fig. S9) despite that the stabilities of phosphine oxide-based compounds are known to be much lower than carbazole-based compounds^33^. Thus, the stability for the radical anion of 2Cz2DMAC2BN might be dominant, which can be explained by the small bond dissociation energy of the C–N bond in the radical anion state^34^.

**Table 2:** OLED performances of 2Cz2DMAC2BN-based devices.

Host	Doping conc. (wt%)	Voltage (V)^a^	EQE (%)^b^	CE (cd A^−1^)^c^	PE (lm W^−1^)^d^	*L*max (cd m^−2^)^e^	λ_EL_/λ_FWHM_ (nm)^f^
mCBP	6	4.0/5.1/6.0	19.0/15.9/11.7	38.5/29.0	38.4/14.6	12,690	495/83
mCBP	15	3.5/4.4/5.4	15.5/15.5/13.5	46.1/39.5	32.9/22.9	19,562	508/82
mCBP	50	3.0/3.7/4.6	15.6/13.2/11.5	44.8/33.7	46.9/23.0	20,657	508/81
CCP	6	2.9/3.3/4.2	10.7/9.0/7.3	30.3/19.8	34.0/14.8	13,340	497/72
CCP	15	2.7/3.1/3.8	17.3/14.8/12.4	44.2/32.8	49.6/27.1	15,123	497/73
CCP	50	2.6/3.0/3.5	15.6/15.3/15.3	46.2/44.5	45.3/38.9	25,641	505/77
PPT	6	3.7/5.2/6.8	17.5/14.4/11.0	53.2/29.1	52.3/13.4	7690	501/83
PPT	15	2.9/3.9/5.0	20.2/17.9/16.0	58.0/45.6	65.0/28.6	16,108	506/82
PPT	50	2.5/3.2/4.3	16.7/16.7/15.5	51.3/47.4	50.4/35.5	20,543	513/84
-	100 (Neat)	2.9/3.5/4.4	13.5/10.7/8.3	41.8/27.0	43.8/19.3	12,302	520/85

^a^Voltage at 1 (turn-on voltage, *V*_on_), 100, and 1000 cd m^−2^, respectively. ^b^EQE for maximum, and at 100, and 1000 cd m^−2^, respectively. ^c^Current Efficiency for maximum, and at 1000 cd m^−2^. ^d^Power Efficiency for maximum, and at 1000 cd m^−2^. ^e^Maximum luminance. ^f^*λ*_EL_: EL emission maximum, and *λ*_FWHM_: full-width at half-maximum.

### Stabilities

**Figure 3:**
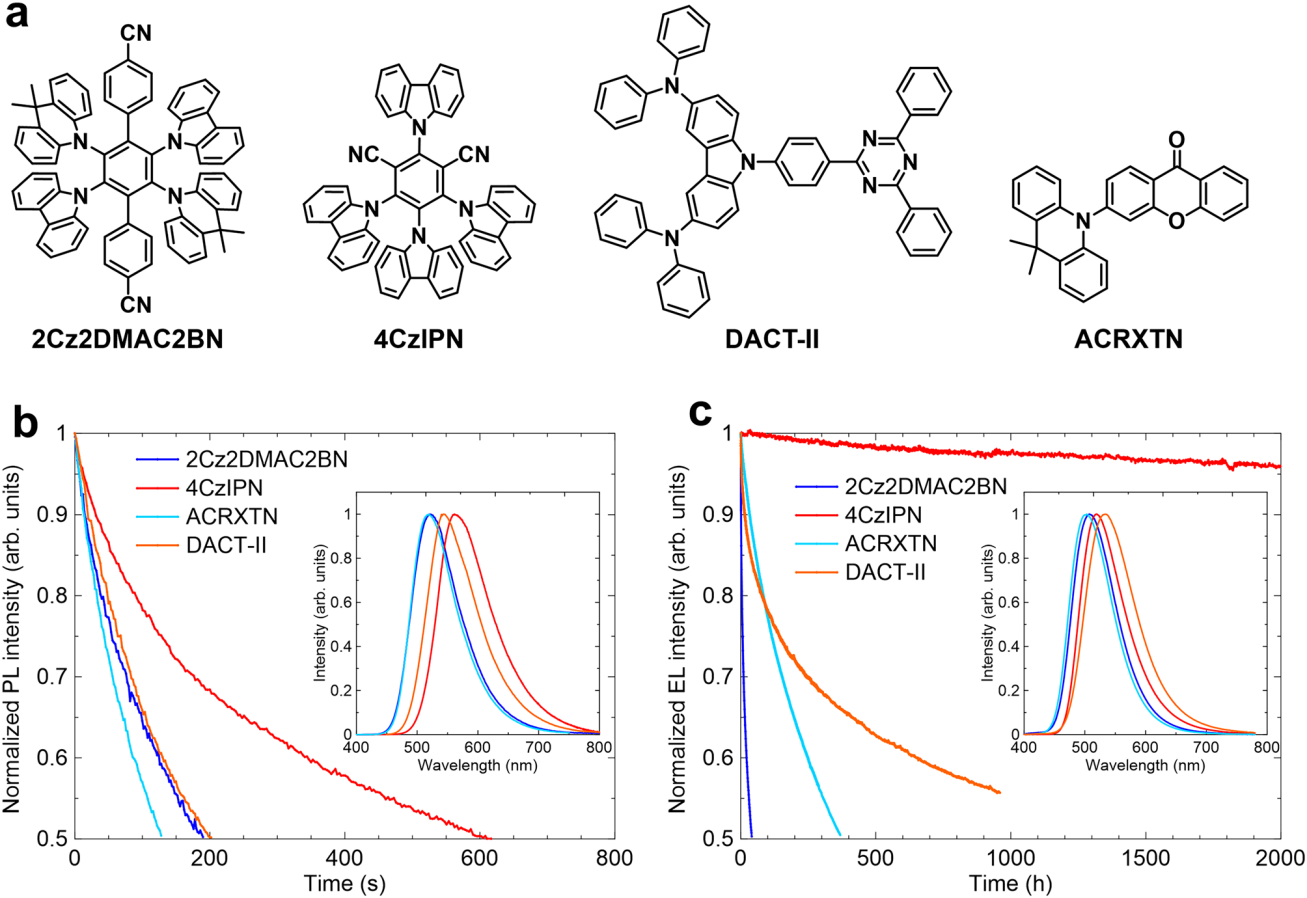
(**a**) Chemical structures of TADF materials under investigation. (**b**) Photostability of TADF materials in the neat films. PL intensity change versus excitation time irradiated by continuous-wave laser light at 355 nm (excitation power of 2.5 W cm^−2^). Inset: PL spectra for the neat films. (**c**) Device operational stability for the OLED having EML of 15 wt%-TADF doped mCBP. Luminance change versus operational time at an initial luminance of 100 cd m^−2^. Inset: EL spectra for the devices.

To obtain a better understanding of the stability issues, we compared four different TADF materials, 2Cz2DMAC2BN, 4CzIPN^1^, ACRXTN^35^, and DACT-II^36^, focusing on the types of the donor structures (Fig. 3). Although 2Cz2DMAC2BN has two types of donors of Cz and DMAC, DMAC moieties provide the HOMO level of the molecule because of the stronger donor nature of DMAC compared to Cz moieties. Thus, the HOMO of 2Cz2DMAC2BN was shallower than that of 4CzIPN (ca. −5.4 eV vs. ca. −5.8 eV) although both molecules have four multiple donor units. ACRXTN having a single DMAC donor has a slightly deeper HOMO energy (ca. −5.7 eV) to 2Cz2DMAC2BN^37^. DACT-II can also be considered to have a single donor unit based on the Cz ring although the HOMO energy (−5.5 eV) is slightly shallower owing to substituents of diphenyl amines at 3,6-positions of carbazole. The photophysical properties of these materials in the neat films were summarized in Supplementary Fig. S10 and Table S3. These compounds showed green emission with similar S_1_ energies. In addition, these materials also have similar τ_d_ values of 1–2 µs and *k*_RISC_ values of 8–10 × 10^5^ s^−1^, which is considered to be an important parameter related to the stability of TADF molecules. However, the excited state stabilities estimated from the PL intensity changes as a function of the irradiation time are largely different for these compounds. The half-life times of the emission intensities are three times longer in 4CzIPN than 2Cz2DMAC2BN and DACT-II, and four times longer compared to ACRXTN. This result cannot be explained by only photophysical parameters such as HOMO–LUMO levels, PLQY, Δ*E*_ST_, and *k*_RISC_ of the materials. Thus, we assume that the stability is strongly affected by the chemical structures of the compounds. Here, we classified two distinct features, that is, multiple donor-based D–A molecules (2Cz2DMAC2BN and 4CzIPN) vs. linearly linked single D–A molecules (ACRXTN and DACT-II), and donor structure types of DMAC-based (2Cz2DMAC2BN and ACRXTN) vs. Cz-based (4CzIPN and DACT-II) molecules. For the same class of donor units, the multiple donor-based D–A molecules showed better stabilities compared to the single linear D–A molecules. This is attributed to the crowded substituents with intramolecular π–π stacking. The photodegraded samples of carbazole derivatives showed products via dissociation of the C–N bond, e.g., carbazole and dicarbazole isophthalonitrile from 4CzIPN^38^. Thus, the delocalization and stabilization of the CT state in the excited state through the donor–donor interactions might suppress the dissociation of the donor units. This may also be related to the results that Cz-based TADF molecules showed better stability than DMAC-based ones. The larger dihedral angles between DMAC and Ph rings should not only weaken the bond strength but also more localize the D–A in the CT. Note that the photostability for the doped films also showed a similar tendency (Supplementary Fig. S11 and Table S4).

We further compared the operational lifetime for the devices based on these four TADF emitters (Fig. 3), where the device structure was ITO (100 nm)/HAT-CN (10 nm)/Tris-PCz (30 nm)/mCBP (EBL) (5 nm)/15-wt%-emitters:mCBP (30 nm)/T2T (10 nm)/BPy-TP2 (40 nm)/Liq (2 nm)/Al (100 nm). Only 4CzIPN showed stable operation and the lifetime is several hundred times longer than those for other materials. Although the triplet formation is huge in the electrical excitation, the *k*_RISC_ of these materials are of the same order of magnitude. Thus, the large difference with the excited state stability suggests that the dominant degradation path in the devices is related to the charged species. Although ACRXTN showed a slightly longer lifetime than 2Cz2DMAC2BN, DMAC moiety seems to essentially cause the lower stability. As mentioned above, the stability of the radical anion state might have a larger impact on the devices of 2Cz2DMAC2BN, and the C–N single bond is known to be vulnerable in the radical anion. Thus, the highly twisted C–N bond between DMAC and Ph rings by the steric hindrance might decrease the stability of 2Cz2DMAC2BN. In the blend films of TADF emitters and commonly used p-type hosts such as mCBP, TADF molecules play a key role in electron injection and transport^39^. Here, for realizing green emission, 2Cz2DMAC2BN, ACRXTN, and DACT-II have relatively strong donors and weak acceptors, while 4CzIPN has relatively weak donors and strong acceptors. These differences indicate that 4CzIPN is advantageous to the balanced charge carrier injection and transport by taking into account the host properties. Thus, the choice of donor and acceptor units should primarily be important and carefully considered.

## Conclusions

A multiple D–A type TADF compound was developed using hetero donors of Cz and DMAC moieties. The fundamental TADF properties were comparable to those of excellent green emitters such as 4CzIPN. In addition, high PLQY can be maintained in the polar solvents and films with high doping concentrations. The OLED performances were optimized by changing the doping concentrations of the emitters, demonstrating the reasonable EQE. The main motivation of the work was to offer a better understanding of the structure-stability relationship. Thus, we compared the stabilities of four different types of TADF materials. The multiple D–A type design seems to be valuable for increasing excited state stability. However, the device operational stability is more complicated, and the polaron-related degradation might have a larger impact on the OLED durability. Since the carrier injection and transport properties in the EML are affected by TADF molecules even though their main function is to control the excitonic process, careful consideration of each donor–acceptor strength in the TADF molecule is necessary.

## Methods

### General

Commercially available materials for the synthesis were used as received from the suppliers. Details of instruments and physical measurements are given in Supplementary Table S6.

### Synthesis and characterizations

The synthetic procedures and characterization data for each material are described in Supplementary Methods S1 and NMR spectra are given in Data S1.

### Quantum calculations

The computations were mainly performed using the computer facilities at the Research Institute for Information Technology, Kyushu University. Molecular orbital calculations were performed using the program Gaussian 16. The geometries were optimized at the B3LYP/6-31+G(d,p). The TD-DFT calculations were conducted at the B3LYP/6-31+G(d,p) level for the excited states calculations.

### Preparation of organic films for photophysical measurements

Organic thin-films for optical measurements were fabricated on clean quartz and silicon substrates by thermal evaporation at a pressure lower than 1 × 10^−4^ Pa. The substrates were cleaned with acetone and isopropanol and then treated with UV/ozone to remove adsorbed organic species before deposition.

### OLED fabrication and performance characterization

The fabrication and measurement procedures are described in Supplementary Methods S2.

### X-ray single crystal analysis

Single crystals suitable for X-ray structural analysis were obtained by vacuum sublimation. X-ray crystallographic information files (CIFs) are available [CCDC 2249866 contains the supplementary crystallographic data for this paper. These data can be obtained free of charge from The Cambridge Crystallographic Data Centre via http://www.ccdc.cam.ac.uk/data_request/cif].

## Supplementary Information

Supplementary Information 1.

Supplementary Information 2.
