# Supplementary material for: Multiple donor–acceptor design for highly luminescent and stable thermally activated delayed fluorescence emitters
Source: Sci Rep. 2023 May 11;13:7644. doi: 10.1038/s41598-023-34623-9 (PMC10175249; doi:10.1038/s41598-023-34623-9)
Supplement: Supplementary file 1 — Supplementary Information 1. [file 41598_2023_34623_MOESM1_ESM.pdf]

Supporting Information

Multiple donor-acceptor design for highly luminescent and stable thermally activated delayed fluorescence emitters

Bhagya Madushani,[1] Masashi Mamada,*[1‡] Kenichi Goushi,[1] Thanh Ba Nguyen,[1] Hajime Nakanotani,[1] Hironori Kaji,[2] Chihaya Adachi*[1,3]

[1] *Center for Organic Photonics and Electronics Research (OPERA), Kyushu University, Fukuoka 819-0395, Japan*

[2] *Institute for Chemical Research, Kyoto University, Uji, Kyoto 611-0011, Japan*

[3] *International Institute for Carbon Neutral Energy Research (WPI-I2CNER), Kyushu University, Nishi, Fukuoka 819-0395, Japan*

[‡] Present address: *Department of Chemistry, Graduate School of Science, Kyoto University, Sakyo-ku, Kyoto 606-8502, Japan*

mamada@kuchem.kyoto-u.ac.jp

adachi@cstf.kyushu-u.ac.jp

Contents:

Methods S1. Synthesis 3

Figure S1. X-ray single crystal analysis 4

Figure S2. Calculated molecular geometries at the B3LYP/6-31+G(d,p) 5

Figure S3. PL spectra in various solvents 5

Table S1. Photophysical characteristics in various solvents 6

Figure S4. PL spectra in CCP and PPT hosts 6

Table S2. Photophysical characteristics in CCP and PPT hosts 7

Figure S5. Energy diagram of the fabricated devices. 7

Figure S6. Electrochemical measurements 8

Figure S7. Radiation patterns from angular dependent PL measurements. 8

Figure S8. Current density-voltage characteristics for HODs and EODs. 9

Figure S9. Luminance change versus operational time for 2Cz2DMAC2BN devices 9

Figure S10. Absorption spectra of the in the neat films of 2Cz2DAMC2BN, 4CzIPN, ACRXTN and DACT-II..... 9

Table S3. Photophysical characteristics of the neat films of 2Cz2DAMC2BN, 4CzIPN, ACRXTN and DACT-II 10

Figure S11. Photostability of TADF materials in the doped films for 2Cz2DMAC2BN and 4CzIPN. 10

Table S4. Photophysical characteristics of the 6 wt%-doped films of 2Cz2DAMC2BN and 4CzIPN in mCBP 10

Table S5. OLED performances of the devices with 15 wt%-TADF emitters doped EML 11

Table S6. Instruments 12

Methods S2. Device structures and fabrications 13

Data S1. NMR spectra 14

Supplementary References 16

Methods S1. Synthesis

9,10-Dihydro-9,9-dimethylacridine (CAS: 6267-02-3) was purchased from Tokyo Kasei Co., Ltd. (D5028, >98.0%). Sodium Hydride, in Oil (CAS: 7646-69-7) was purchased from WAKO Chemical Industries (191-07662). Carbazole (86-74-8) was purchased from USHIO Chemix Co., Ltd.

Synthesis scheme 1

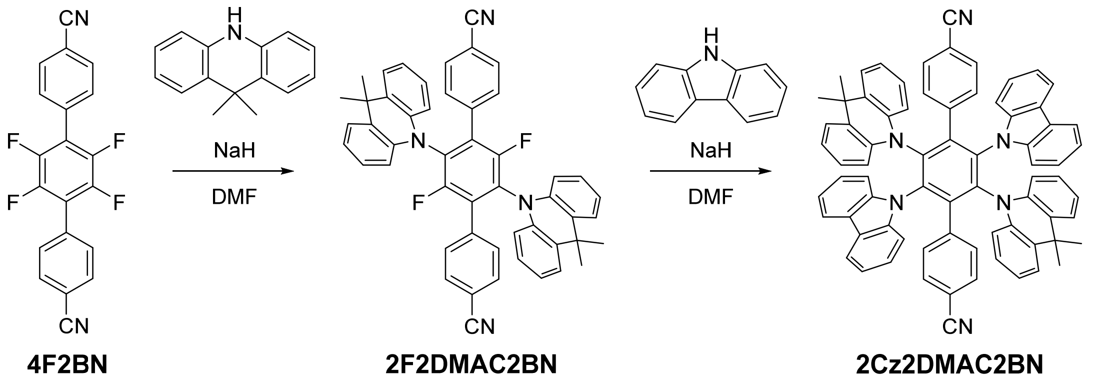

2',3',5',6'-Tetrafluoro-[1,1':4',1''-terphenyl]-4,4''-dicarbonitrile (**4F2BN**) was synthesized by modifying the literature.[S1]

Compound **2F2DMAC2BN**

To a dispersion of sodium hydride (60% in mineral oil, 0.38 g, 9.5 mmol) in dry DMF (15 ml) was added 9,10-dihydro-9,9-dimethylacridine (1.89 g, 9.1 mmol) in DMF (15 ml) at 0 °C under Ar. After stirring for 30 min, **4F2BN** (0.88 g, 2.5 mmol) was added as a solid. After stirring for 30 min, the reaction mixture was stirred at 80 °C overnight. After cooling, H_2O was added and the precipitates were filtered, washed with H_2O and methanol. The crude product was purified by sublimation at 300 °C under vacuum, then column chromatography on a silica gel using DCM (R_f = 0.61), and recrystallization from DCM layered with MeOH to give pure compound as a yellow solid (1.10 g, 60%). ^{1}H-NMR: δ/ppm (500 MHz, $CDCl_3$, Me_4Si) = 7.48 (d, 4H, J = 8.0 Hz); 7.45 (d, 4H, J = 8.0 Hz); 7.35 (d, 4H, J = 8.0 Hz); 7.17 (dd, 4H, J = 7.5 Hz, J = 8.0 Hz); 7.02 (dd, 4H, J = 7.5 Hz, J = 8.0 Hz); 6.44 (d, 4H, J = 8.0 Hz); 1.80 (s, 6H); 1.05 (s, 6H). ^{19}F NMR (470 MHz, $CDCl_3$, 300K, δ): −118.4. ^{13}C-NMR: δ/ppm (125 MHz, $CDCl_3$) = 156.8, 154.7, 138.8, 134.9, 132.7, 132.0, 130.4, 130.4, 128.4, 127.2, 126.4, 122.2, 118.3, 113.0, 112.4, 35.9, 34.7, 29.2. MS (ASAP) m/z (%): 731 (100) [$M+1^+$]. Anal. Calcd. For $C_{50}H_{36}F_2N_4$: C, 82.17; H, 4.97; F, 5.20; N, 7.67. Found: C, 82.12; H, 5.03; N, 7.65.

Compound **2Cz2DMAC2BN**

To a dispersion of sodium hydride (60% in mineral oil, 0.53 g, 13.4 mmol) in dry DMF (15 ml) was added carbazole (2.20 g, 13.2 mmol) in DMF (15 ml) at 0 °C under Ar. After stirring for 30 min, **2F2DMAC2BN** (1.20 g, 1.6 mmol) was added as a solid. After stirring for 30 min, the reaction mixture was stirred at 80 °C overnight. After cooling, H_2O was added and the precipitates were filtered, washed with H_2O and methanol. The crude product was purified by sublimation at 380 °C under vacuum to remove carbazole, then column chromatography on a silica gel using the hexane/DCM (1:2) mixture as eluent (R_f = 0.32), recrystallization from DCM layered with MeOH, and sublimation at 380 °C to give pure compound as a yellow solid (1.40 g, 83%).

^{1}H-NMR: δ/ppm (500 MHz, $CDCl_3$, Me_4Si) = 7.69 (d, 4H, J = 6.4 Hz); 7.03 (d, 4H, J = 8.4 Hz); 7.00–6.98 (m, 16H); 6.89 (dd, 4H, J = 8.0 Hz); 6.79 (d, 4H, J = 8.4 Hz); 6.69–6.64 (m, 8H); 1.33 (s, 6H); 0.42 (s, 6H). ^{13}C-NMR: δ/ppm (125 MHz, $CDCl_3$) = 148.5, 140.2, 139.9, 139.1, 138.4, 136.9, 130.9, 129.8, 129.4, 127.0, 126.0, 125.1, 123.3, 121.3, 120.2, 120.1, 118.4, 114.1, 111.9, 110.7, 35.0, 34.9, 32.7. MS (ASAP) m/z (%): 1026 (100) [M+1$^+$]. Anal. Calcd. For $C_{74}H_{52}N_6$: C, 86.69; H, 5.11; N, 8.20. Found: C, 86.65; H, 5.16; N, 8.13.

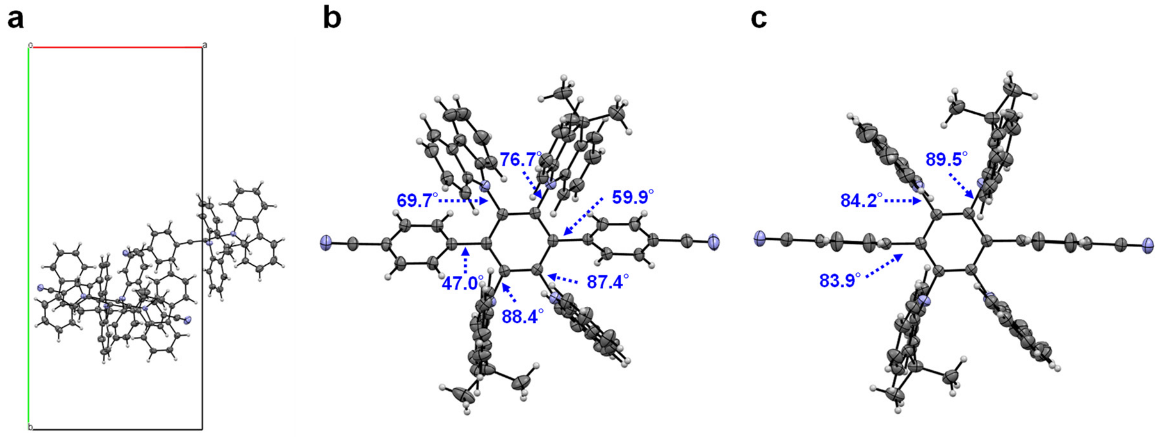

Figure S1. X-ray single crystal analysis. (a) The molecular structure in the asymmetric unit. (b) The dihedral angles for molecule 1. (c) The dihedral angles for molecule 2.

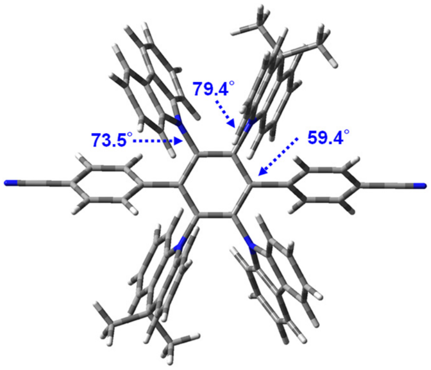

Figure S2. Calculated molecular geometries at the B3LYP/6-31+G(d,p).

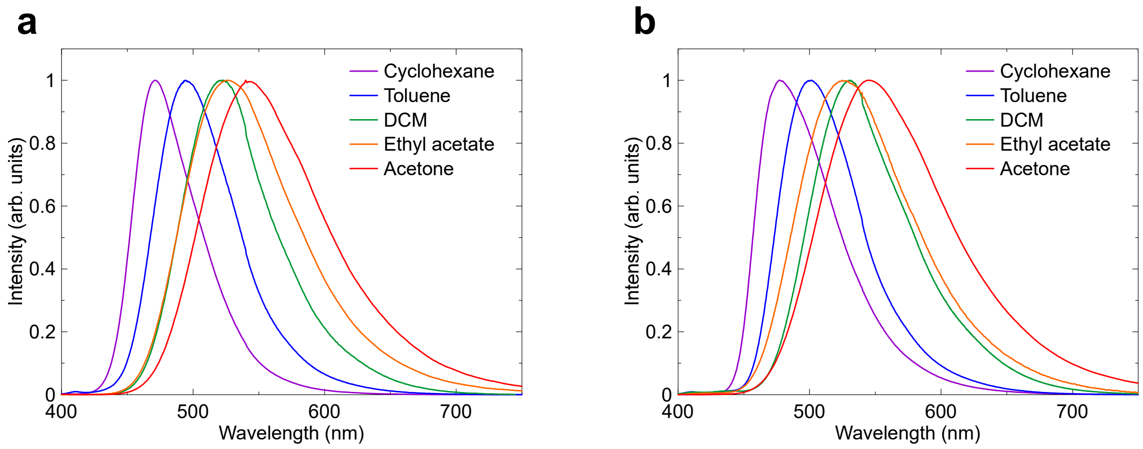

Figure S3. PL spectra in various solvents for (a) 2Cz2DMAC2BN and (b) 4CzIPN

Table S1. Photophysical characteristics in various solvents for (a) 2Cz2DMAC2BN and (b) 4CzIPN

Compound	Solvents	λ_{PL} [nm]	S_1 [eV]	T_1 [eV]	ΔE_{ST} [eV]	Φ_{PL} [-][a)]	τ_d [μs][a)]
2Cz2DMAC2BN	In cyclohexane	472	2.85	2.80	0.05	0.51	5.4
	In toluene	495	2.79	2.78	0.01	0.91	3.8
	In dichloromethane	522	2.71	2.70	0.01	0.73	4.4
	In ethyl acetate	526	2.73	2.73	0	0.70	4.2
	In acetone	544	2.66	2.66	0	0.68	1.2
4CzIPN	In cyclohexane	478	2.78	2.63	0.15	0.90	4.8
	In toluene	501	2.71	2.67	0.04	0.81	4.7
	In dichloromethane	530	2.66	2.64	0.02	0.59	4.4
	In ethyl acetate	526	2.69	2.66	0.03	0.59	4.2
	In acetone	546	2.65	2.63	0.02	0.41	3.6

[a)]Measured under N_2 in solution

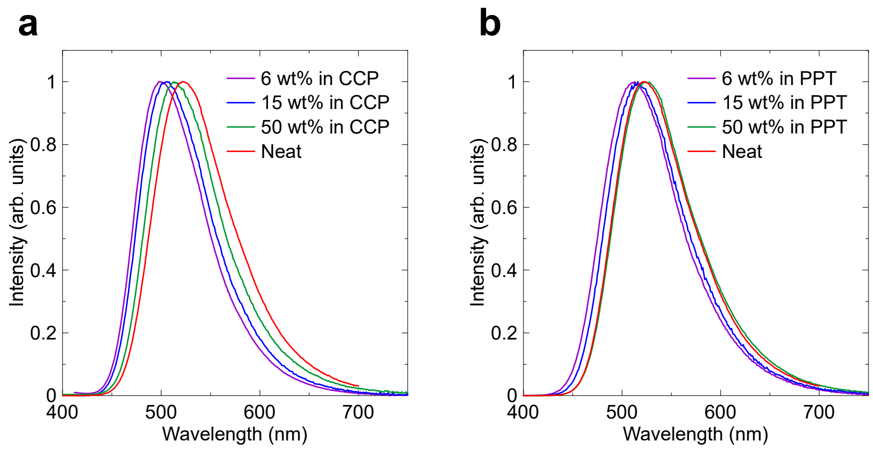

Figure S4. PL spectra in CCP and PPT hosts for 2Cz2DMAC2BN

Table S2. Photophysical characteristics in CCP and PPT hosts for 2Cz2DMAC2BN

Condition	λ_{PL} [nm]	S_1 [eV]	T_1 [eV]	ΔE_{ST} [eV]	Φ_{PL} [-][a)]	Φ_p/Φ_d [-][a)]	τ_p [ns][a)]	τ_d [μs][a)]	k_r [10^6 s^{-1}]	k_{ISC} [10^7 s^{-1}]	k_{RISC} [10^5 s^{-1}]
In 6 wt% film of CCP	500	2.77	2.76	0.01	0.89	0.29/0.60	39	3.8	7.4	1.8	7.7
In 15 wt% film of CCP	505	2.75	2.73	0.02	0.91	0.28/0.63	49	3.3	5.7	1.5	9.5
In 50 wt% film of CCP	514	2.67	2.66	0.01	0.96	0.36/0.60	53	3.1	6.8	1.2	8.4
In 6 wt% film of PPT	511	2.75	2.73	0.02	0.89	0.38/0.51	58	2.4	6.5	1.1	9.0
In 15 wt% film of PPT	516	2.72	2.70	0.02	0.99	0.43/0.56	67	2.6	6.4	0.85	8.8
In 50 wt% film of PPT	525	2.66	2.62	0.04	0.90	0.47/0.43	58	3.5	8.1	0.91	4.9

a)Measured under Ar.

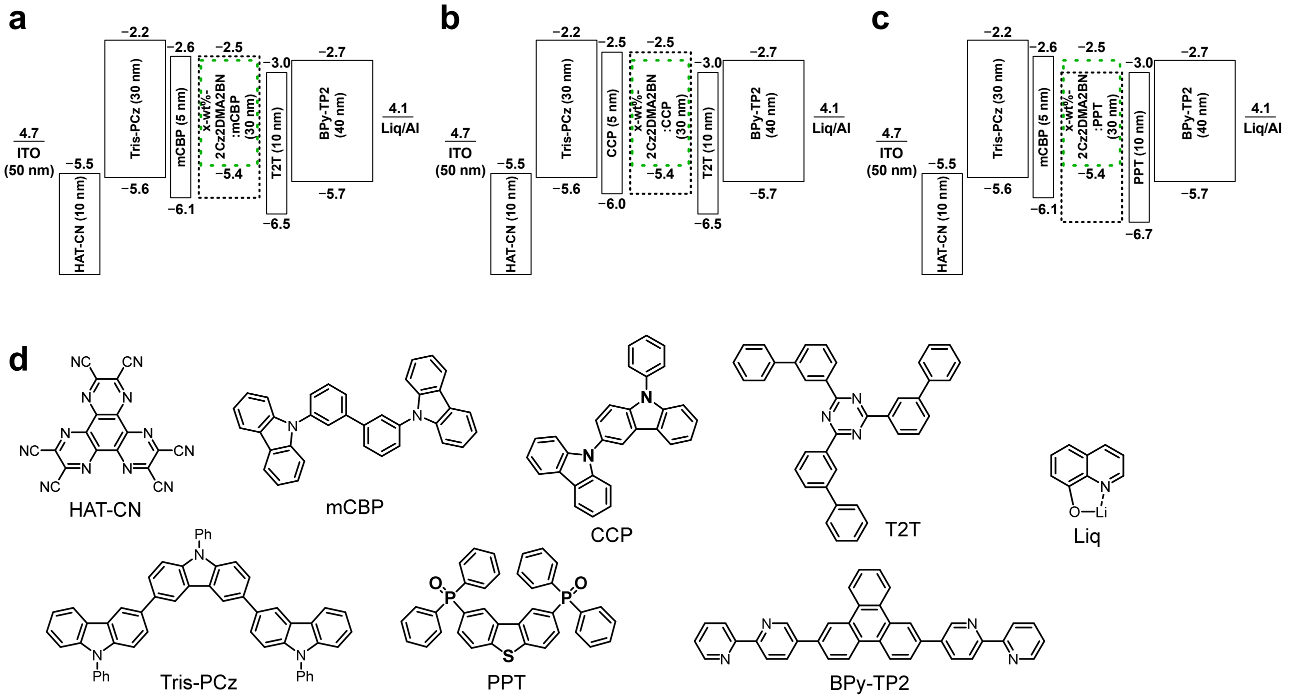

Figure S5. Energy diagram of the fabricated devices. (a) mCBP host. (b) CCP host. (c) PPT host. (d) Chemical structures of the materials. The compound name are as follows: HATCN (dipyrazino[2,3-*f*:2',3'-*h*]quinoxaline-2,3,6,7,10,11-hexacarbonitrile), tris-PCz (9,9',9''-triphenyl-9*H*,9'*H*,9''*H*-3,3':6',3''-tercarbazole), T2T (2,4,6-tris(biphenyl-3-yl)-1,3,5-triazine), BPy-TP2 (2,7-bis(2,2'-bipyridine-5-yl)triphenylene), and Liq (8-hydroxyquinolinolato-lithium).

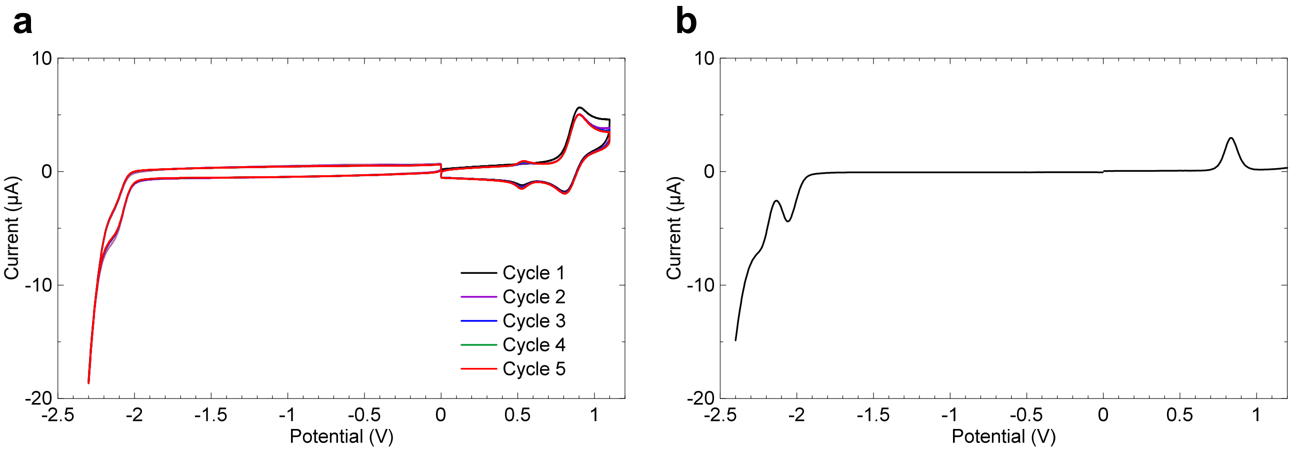

Figure S6. Electrochemical measurements in dichloromethane. (a) Cyclic voltammograms (CV) (vs Ag/Ag^{+}). (b) Differential pulse voltammograms (DPV) (vs Ag/Ag^{+})

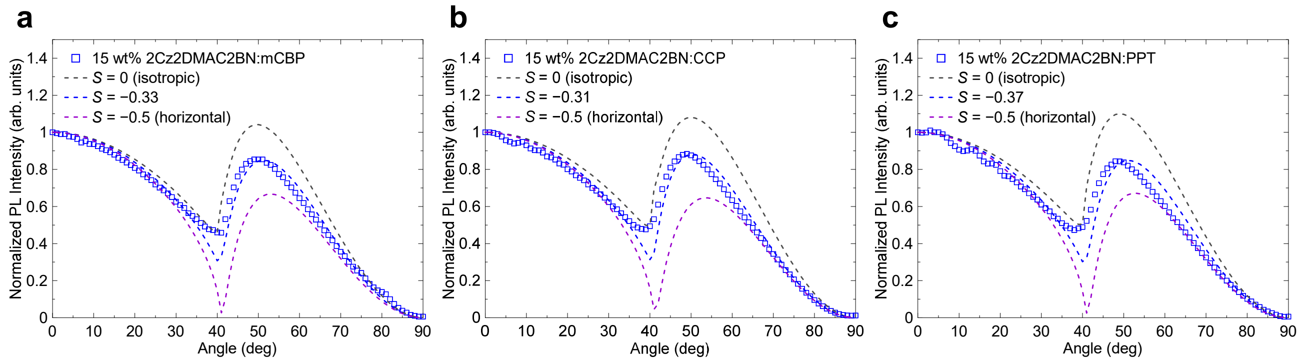

Figure S7. Radiation patterns from angular dependent PL measurements. (a) 15 nm thick film of 2Cz2DMAC2BN doped film in (a) mCBP, (b) CCP, and (c) PPT. The optical simulation was performed using simulation software (Setfos 4.6, Fluxim).[S2] The n and k values for each layer were measured by variable angle spectroscopic ellipsometry.

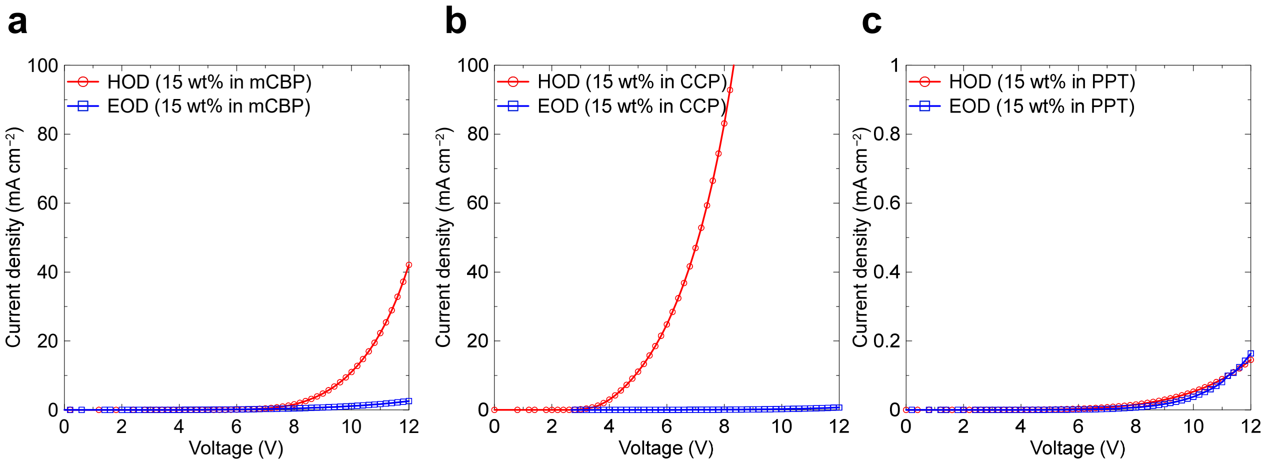

Figure S8. Current density-voltage characteristics for HODs and EODs. The devices with the EML of 15 wt%-2Cz2DMAC2BN-doped (a) mCBP, (b) CCP, and (c) PPT.

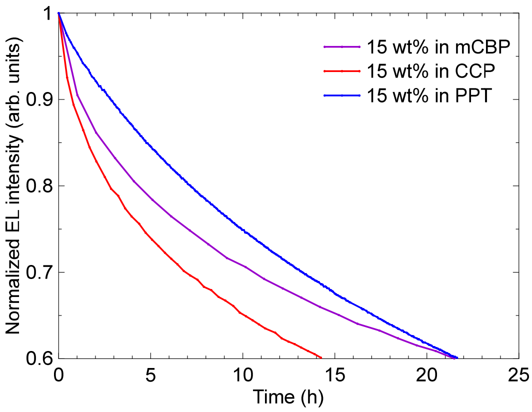

Figure S9. Luminance change versus operational time for 2Cz2DMAC2BN devices at an initial luminance of 100 cd m^{-2}.

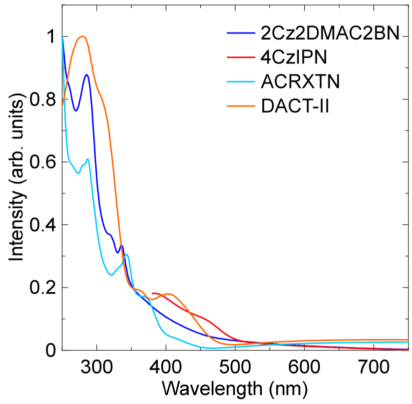

Figure S10. Absorption spectra of the in the neat films of 2Cz2DAMC2BN, 4CzIPN, ACRXTN and DACT-II.

Table S3. Photophysical characteristics of the neat films of 2Cz2DAMC2BN, 4CzIPN, ACRXTN and DACT-II

Compound	λ_{PL} [nm]	S_1 [eV]	T_1 [eV]	ΔE_{ST} [eV]	Φ_{PL} [-][a)]	τ_p [ns][a)]	τ_d [μs][a)]	k_r [10^6 s^{-1}]	k_{ISC} [10^7 s^{-1}]	k_{RISC} [10^5 s^{-1}]	E_g^{opt} [eV]
2Cz2DMAC2BN	522	2.67	2.60	0.07	0.76	62	2.0	6.1	1.0	8.1	2.6
4CzIPN	563	2.45	2.40	0.05	0.43	22	1.3	11	3.5	8.1	2.4
ACRXTN	519	2.67	2.61	0.06	0.91	46	1.9	9.8	1.2	9.8	2.7
DACT-II	544	2.54	2.43	0.11	0.44	62	2.0	3.2	1.3	7.8	2.6

[a)]Measured under Ar

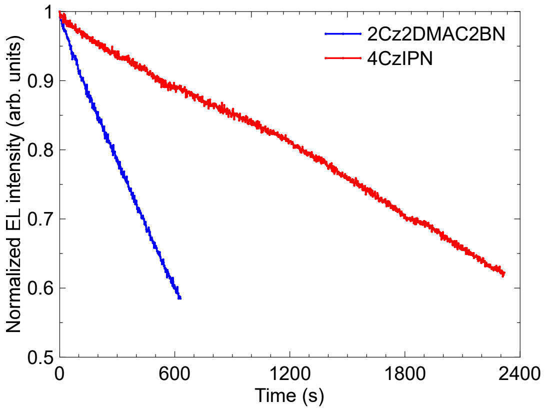

Figure S11. Photostability of TADF materials in the doped films for 2Cz2DMAC2BN and 4CzIPN. PL intensity change versus excitation time irradiated by continuous-wave laser light at 355 nm (excitation power of 2.7 W cm^{-2})

Table S4. Photophysical characteristics of the 6 wt%-doped films of 2Cz2DAMC2BN and 4CzIPN in mCBP

Compound	λ_{PL} [nm]	S_1 [eV]	T_1 [eV]	ΔE_{ST} [eV]	Φ_{PL} [-][a)]	Φ_p/Φ_d [-][a)]	τ_p [ns][a)]	τ_d [μs][a)]	k_r [10^6 s^{-1}]	k_{ISC} [10^7 s^{-1}]	k_{RISC} [10^5 s^{-1}]
2Cz2DMAC2BN	498	2.76	2.71	0.05	0.95	0.28/0.67	34	3.6	8.2	2.1	9.4
4CzIPN	520	2.70	2.67	0.03	0.93	0.23/0.70	18	2.6	12.9	4.3	14.9

[a)]Measured under Ar

Table S5. OLED performances of the devices with 15 wt%-TADF emitters doped EML

TADF emitters	λ_{EL} [nm]	S_1 [eV]	Voltage [V][a)]	EQE [%][b)]
2Cz2DMAC2BN	508	2.72	3.5/4.4/5.4	15.5/15.5/13.5
4CzIPN	521	2.64	3.2/4.2/5.0	16.9/16.3/15.2
ACRXTN	502	2.73	3.2/4.1/5.3	10.2/10.4/10.2
DACT-II	533	2.63	3.2/3.8/4.8	17.2/15.5/12.5

[a)]Voltage at 1 (turn-on voltage, V_{on}), 100, and 1000 cd m^{-2}, respectively. [b)]EQE for maximum, and at 100, and 1000 cd m^{-2}, respectively.

Table S6. Instruments

Instruments	Brands and Types	Conditions
Sublimation	ALS technology	Three-zone sublimation apparatus.
Elemental Analysis	Yanaco MT-5 CHN corder	
MS	Waters 3100 Mass Spectrometer	Direct probe ionization, ASAP-MS.
NMR	Bruker AVANCE III 500 MHz spectrometer	Chemical shifts were calibrated to the corresponding deuterated solvents.
UV-Vis	Perkin-Elmer Lambda 950-PKA UV–vis spectrophotometer	The light source consisted of Deuterium (D2) and Tungsten Iodide (50W) lamps for the ultraviolet and visible regions.
PL	JASCO FP-8600 fluorometer	the excitation wavelength was set to the absorption maximum
PL quantum Yield	Hamamatsu Photonics Quantaurus-QY C11347-01	Absolute PL quantum yield. The measurement error for the obtained values on this instrument is ±3%.
Spin-coater	Active Co., Ltd., ACT-220D II	800 rpm, 60 s at room temperature
Transient photo-luminescence decay	Hamamatsu Photonics Quantaurus-Tau C11367-03	
UV/ozone	Nippon Laser & Electronics Lab. NL-UV253	15 min
Variable-angle spectroscopic ellipsometry (VASE)	J.A. Wollam, M-2000U	Different angles from 45° to 75° by steps of 5°<br>Analytical software: J.A. Woollam, WVASE32.
Surface profiler	Bruker Dektak XT	A tip radius of 12.5 μm and a scan resolution of 0.168 μm/point.
CV/DPV	BAS 608D + DPV Electrochemical system	Supporting electrolyte: tetrabutylammonium perchlorate (TBAP) (0.1 M) in acetonitrile.<br>Working electrode: an ITO, Auxiliary electrode: a platinum wire, Reference electrode: an $Ag/AgNO_3$.<br>Calibration: ferrocenium-ferrocene (Fc/Fc^+) as a standard.<br>Scan rate: 100 mV s^{-1} for cyclic voltammograms.
Photoelectron yield spectroscopy (PYS)	Riken Keiki, AC-3	in air
TG-DTA	Bruker TG-DTA 2400SA,	Under a N_2 atmosphere or 1 Pa with a heating rate of 10 °C per minute.
DSC	NETZSCH DSC 204F1 Phoenix	20 °C per minute.

Methods S2. Device structures and fabrications

Glass substrates with a prepatterned, 100 nm thick ITO coating were used as anodes. Substrates were washed by sequential ultrasonication in acetone, Semico Clean, distilled water, and isopropanol, dried by isopropanol vapor, and then, ex-posed to UV/ozone. Organic layers were formed by thermal evaporation at a pressure lower than 1×10^{-4} Pa. After fabrication, the devices were immediately encapsulated with glass lids using epoxy glue in a nitrogen-filled glove box (O_2 about 0.1 ppm, H_2O about 0.1 ppm). The current-density–voltage–luminance (*J*–*V*–*L*) characteristics of the OLEDs were evaluated using a source meter (Keysight B2911A, Keysight Technologies) and a luminance meter (CS-2000, Konica Minolta, Japan) at a constant DC current at room temperature. The reproducibility of the device performance of the presented devices was confirmed by measuring at least four different samples. For the device lifetime tests, the luminance and electroluminescence spectra of the driving devices in the normal direction were measured using a luminance meter (SR-3AR, TOPCON, Japan) under constant current density driving conditions with an initial luminance of 100 cd m^{-2}.

Data S1. NMR spectra

Compound **2F2DMAC2BN**

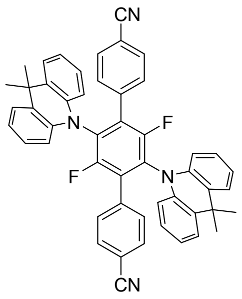

^{1}H-NMR spectrum (500 MHz, $CDCl_3$, 300 K)

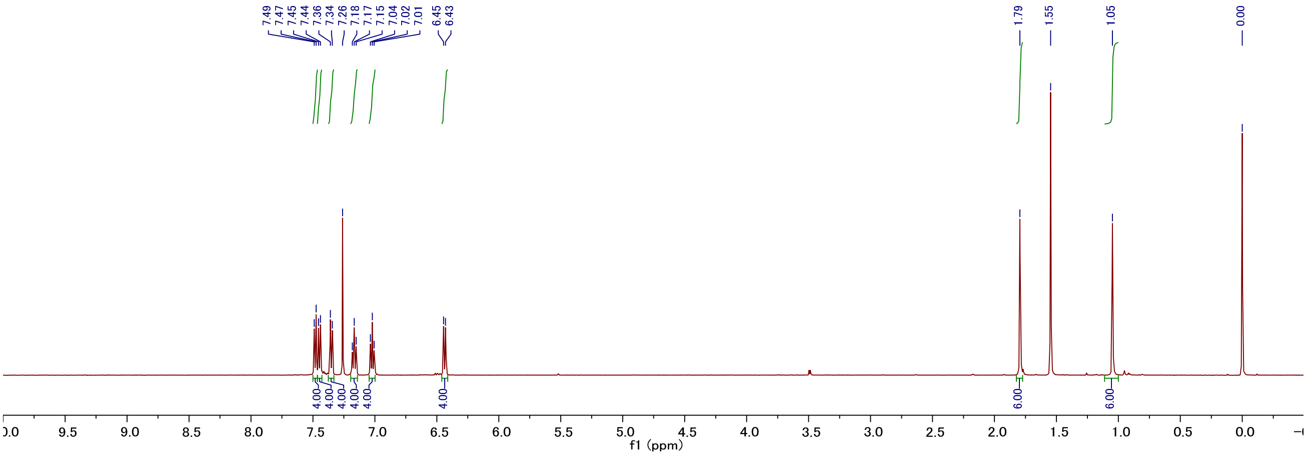

^{19}F-NMR spectrum (470 MHz, $CDCl_3$, 300 K)

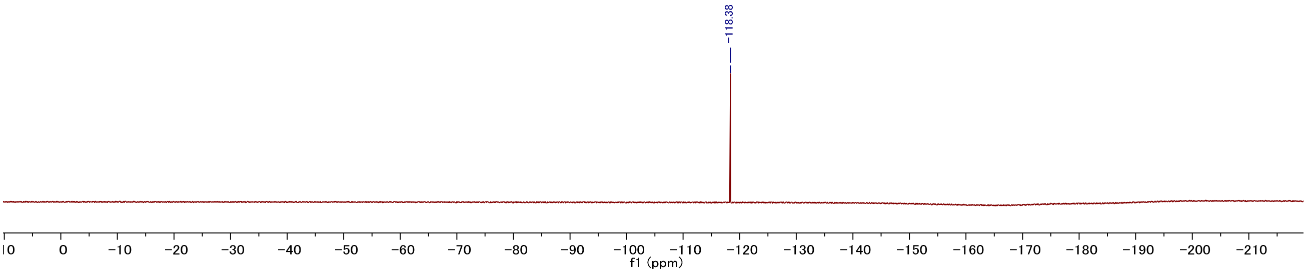

^{13}C-NMR-APT spectra (125 MHz, $CDCl_3$, 300 K)

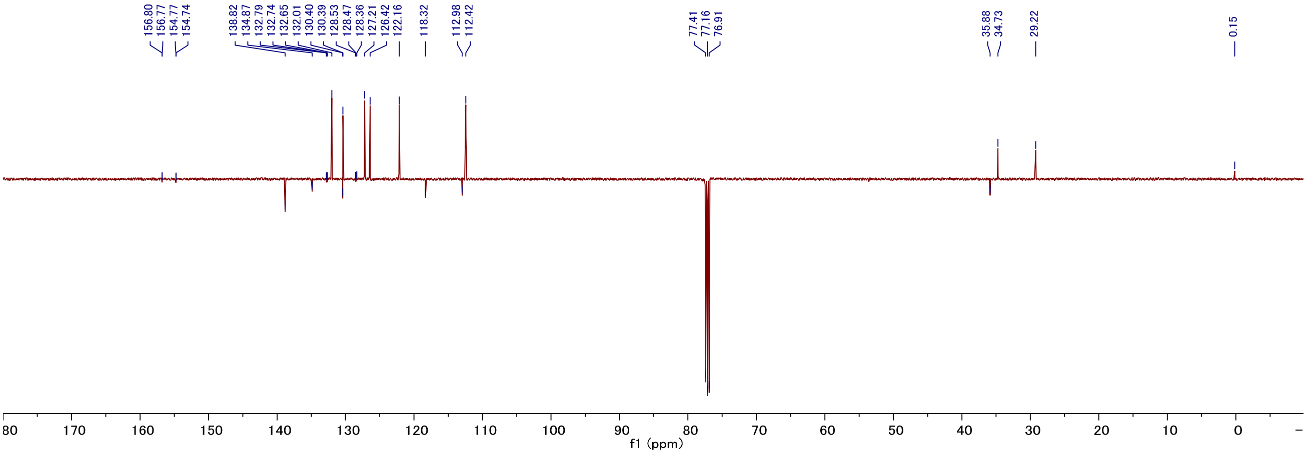

Compound **2Cz2DMAC2BN**

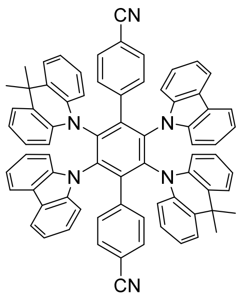

^{1}H-NMR spectrum (500 MHz, $CDCl_3$, 300 K)

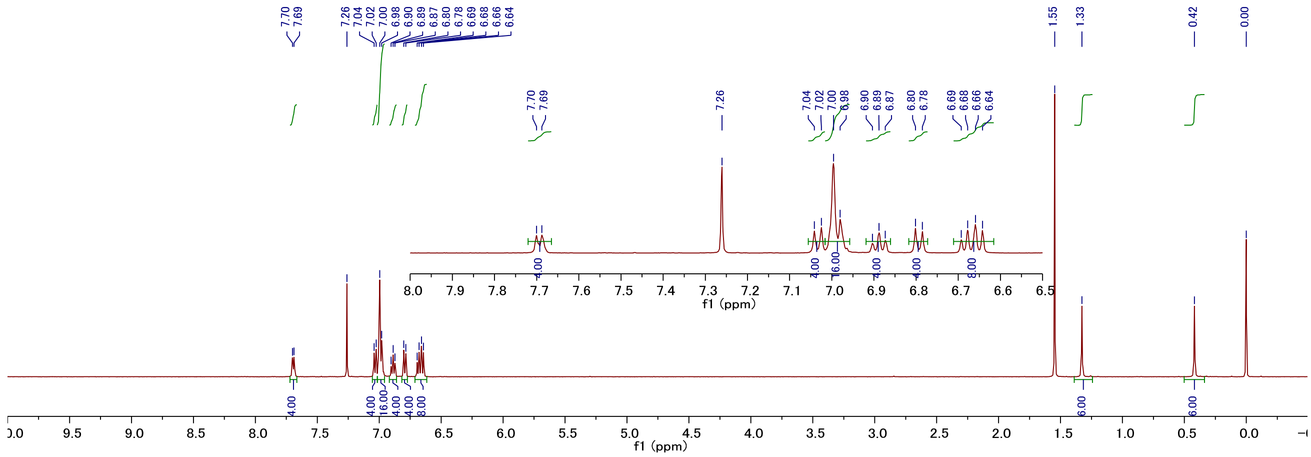

^{13}C-NMR-APT spectra (125 MHz, $CDCl_3$, 300 K)

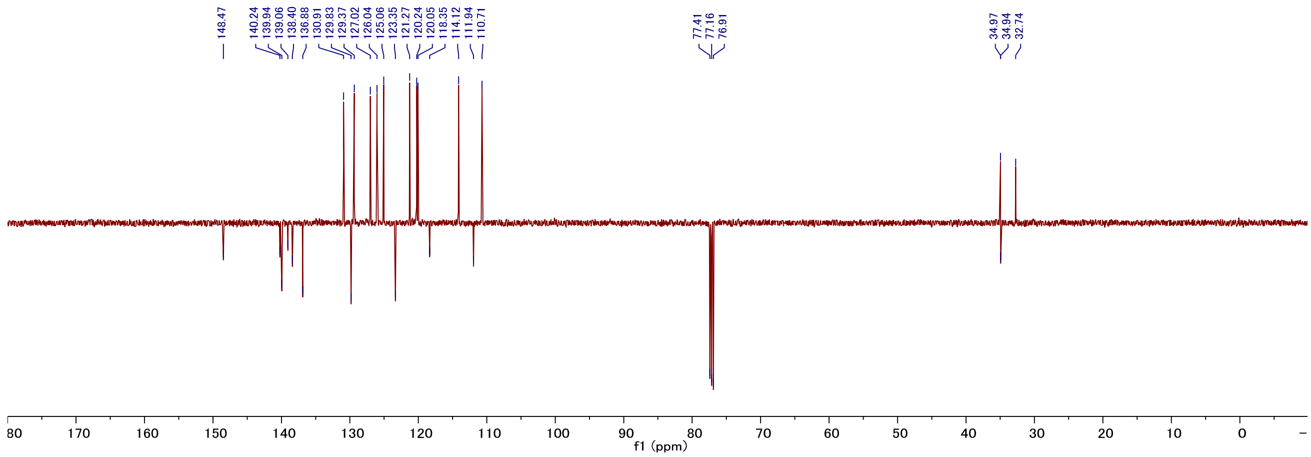

Supplementary References

(S1) Mamada, M. *et al.* Highly efficient deep-blue organic light-emitting diodes based on rational molecular design and device engineering. *Adv. Funct. Mater.* **32,** 2204352 (2022).
(S2) Brütting, W., Frischeisen, J., Schmidt, T. D., Scholz, B. J. & Mayr, C. Device efficiency of organic light-emitting diodes: progress by improved light outcoupling. *Phys. Status Solidi A* **210,** 44−65 (2013).
