# Supplementary material for: Multiple donor–acceptor design for highly luminescent and stable thermally activated delayed fluorescence emitters
Source: Sci Rep. 2023 May 11;13:7644. doi: 10.1038/s41598-023-34623-9 (PMC10175249; doi:10.1038/s41598-023-34623-9)

checkCIF/PLATON report

You have not supplied any structure factors. As a result the full set of tests cannot be run.

THIS REPORT IS FOR GUIDANCE ONLY. IF USED AS PART OF A REVIEW PROCEDURE FOR PUBLICATION, IT SHOULD NOT REPLACE THE EXPERTISE OF AN EXPERIENCED CRYSTALLOGRAPHIC REFEREE.

No syntax errors found. CIF dictionary Interpreting this report

Datablock: 2Cz2DMAC2BN

```
Bond precision:   C-C = 0.0033 A          Wavelength=1.54184

Cell:            a=15.7505(3)    b=32.4973(4)          c=17.2987(2)
                 alpha=90        beta=110.1794(17)     gamma=90
Temperature:     100 K

                 Calculated                    Reported
Volume           8310.8(2)                     8310.8(2)
Space group      P 21/n                        P 1 21/n 1
Hall group       -P 2yn                        -P 2yn
Moiety formula   C74 H52 N6                    C74 H52 N6
Sum formula      C74 H52 N6                    C74 H52 N6
Mr               1025.22                       1025.21
Dx,g cm-3        1.229                         1.229
Z                6                             6
Mu (mm-1)        0.558                         0.558
F000             3228.0                        3228.0
F000'            3236.59
h,k,lmax         19,40,21                      19,40,21
Nref             17248                         17007
Tmin,Tmax        0.919,0.948                   0.647,1.000
Tmin'            0.898

Correction method= # Reported T Limits: Tmin=0.647 Tmax=1.000
AbsCorr = GAUSSIAN

Data completeness= 0.986           Theta(max)= 75.556

R(reflections)= 0.0732( 15273)                          wR2(reflections)=
                                                        0.2060( 17007)
S = 1.100                  Npar= 1087
```

The following ALERTS were generated. Each ALERT has the format
test-name_ALERT_alert-type_alert-level.
Click on the hyperlinks for more details of the test.

Alert level C

```
PLAT761_ALERT_1_C CIF Contains no X-H Bonds ......................     Please Check
PLAT762_ALERT_1_C CIF Contains no X-Y-H or H-Y-H Angles .........     Please Check
```

Alert level G

```
PLAT072_ALERT_2_G SHELXL First  Parameter in WGHT  Unusually Large       0.12 Report
PLAT802_ALERT_4_G CIF Input Record(s) with more than 80 Characters          1 Info
PLAT933_ALERT_2_G Number of HKL-OMIT Records in Embedded .res File          8 Note
PLAT941_ALERT_3_G Average HKL Measurement Multiplicity ..........         4.8 Low
```

0 **ALERT level A** = Most likely a serious problem - resolve or explain
0 **ALERT level B** = A potentially serious problem, consider carefully
2 **ALERT level C** = Check. Ensure it is not caused by an omission or oversight
4 **ALERT level G** = General information/check it is not something unexpected

2 ALERT type 1 CIF construction/syntax error, inconsistent or missing data
2 ALERT type 2 Indicator that the structure model may be wrong or deficient
1 ALERT type 3 Indicator that the structure quality may be low
1 ALERT type 4 Improvement, methodology, query or suggestion
0 ALERT type 5 Informative message, check

Publication of your CIF

You should attempt to resolve as many as possible of the alerts in all categories. Often the minor alerts point to easily fixed oversights, errors and omissions in your CIF or refinement strategy, so attention to these fine details can be worthwhile. In order to resolve some of the more serious problems it may be necessary to carry out additional measurements or structure refinements. However, the nature of your study may justify the reported deviations from journal submission requirements and the more serious of these should be commented upon in the discussion or experimental section of a paper or in the "special_details" fields of the CIF. *checkCIF* was carefully designed to identify outliers and unusual parameters, but every test has its limitations and alerts that are not important in a particular case may appear. Conversely, the absence of alerts does not guarantee there are no aspects of the results needing attention. It is up to the individual to critically assess their own results and, if necessary, seek expert advice.

If you wish to submit your CIF for publication in Acta Crystallographica Section C or E, you should upload your CIF via the web. If you wish to submit your CIF for publication in IUCrData you should upload your CIF via the web. If your CIF is to form part of a submission to another IUCr journal, you will be asked, either during electronic submission or by the Co-editor handling your paper, to upload your CIF via our web site.

PLATON version of 12/09/2022; check.def file version of 09/08/2022

Datablock 2Cz2DMAC2BN - ellipsoid plot

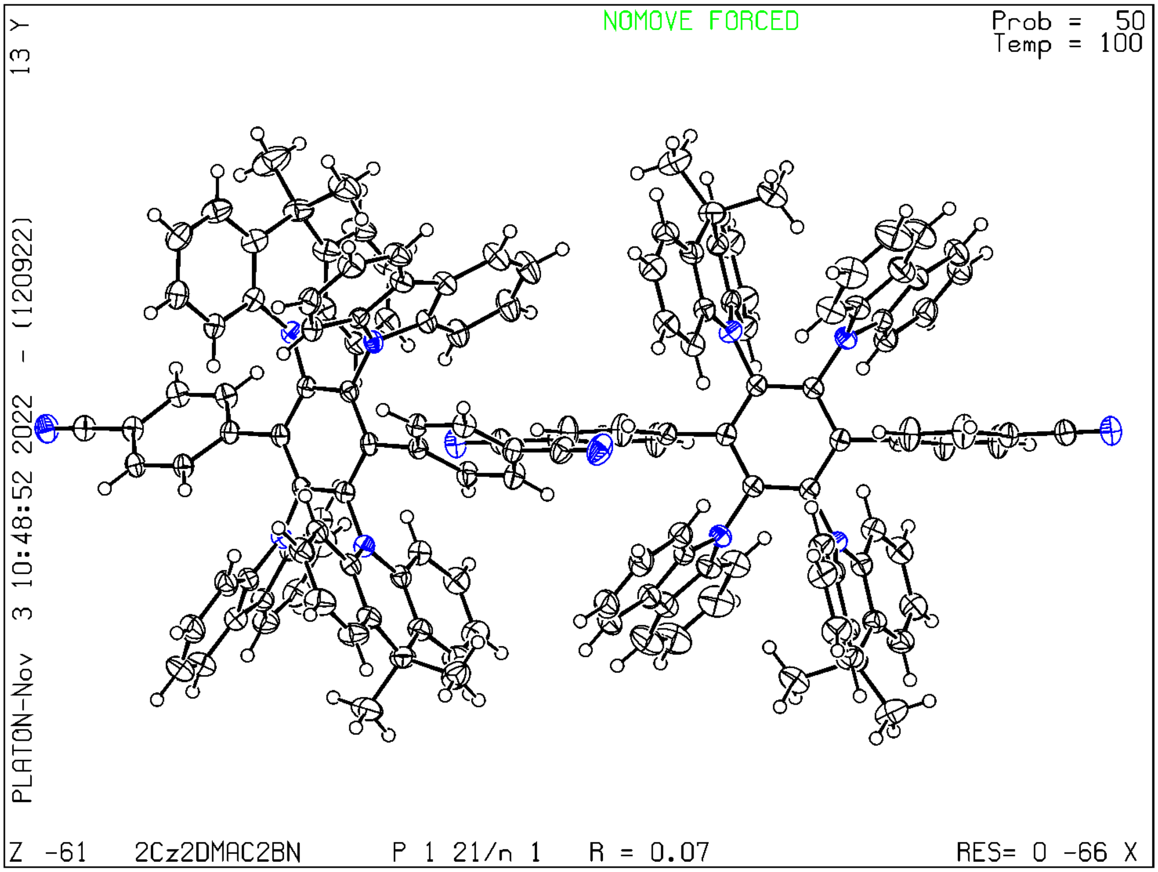

Supplement: Supplementary file 2 — Supplementary Information 2. [file 41598_2023_34623_MOESM2_ESM.pdf]
